# Comparative analysis of image classification methods for automatic diagnosis of ophthalmic images

**DOI:** 10.1038/srep41545

**Published:** 2017-01-31

**Authors:** Liming Wang, Kai Zhang, Xiyang Liu, Erping Long, Jiewei Jiang, Yingying An, Jia Zhang, Zhenzhen Liu, Zhuoling Lin, Xiaoyan Li, Jingjing Chen, Qianzhong Cao, Jing Li, Xiaohang Wu, Dongni Wang, Wangting Li, Haotian Lin

**Affiliations:** 1 Institute of Software Engineering, Xidian University, Xi’an 710071, China; 2 School of Computer Science and Technology, Xidian University, Xi’an 710071, China; 3 School of Software, Xidian University, Xi’an 710071, China; 4 State Key Laboratory of Ophthalmology, Zhongshan Ophthalmic Center, Sun Yat-sen University, Guangzhou 510060, China

## Abstract

There are many image classification methods, but it remains unclear which methods are most helpful for analyzing and intelligently identifying ophthalmic images. We select representative slit-lamp images which show the complexity of ocular images as research material to compare image classification algorithms for diagnosing ophthalmic diseases. To facilitate this study, some feature extraction algorithms and classifiers are combined to automatic diagnose pediatric cataract with same dataset and then their performance are compared using multiple criteria. This comparative study reveals the general characteristics of the existing methods for automatic identification of ophthalmic images and provides new insights into the strengths and shortcomings of these methods. The relevant methods (local binary pattern +SVMs, wavelet transformation +SVMs) which achieve an average accuracy of 87% and can be adopted in specific situations to aid doctors in preliminarily disease screening. Furthermore, some methods requiring fewer computational resources and less time could be applied in remote places or mobile devices to assist individuals in understanding the condition of their body. In addition, it would be helpful to accelerate the development of innovative approaches and to apply these methods to assist doctors in diagnosing ophthalmic disease.

The diagnosis of ocular diseases mainly depends on the observation of various ophthalmic images from patients. Numerous studies have been reported on computer-aided diagnosis of ophthalmic diseases^1^,^2^,^3^,^4^,^5^,^6^,^7^,^8^,^9^,^10^,^11^,^12^,^13^. However, not all image classification methods are suitable for automatic diagnosis of ocular disease. Therefore, it is imperative to compare the feasibility and performance of image classification methods in diagnosing ophthalmic diseases, which could facilitate the follow-up studies on the automatic diagnosis of ophthalmic diseases and shed a light on its application.

Slit-lamp image is an important kind of ophthalmic image and thus some diseases can be diagnosed using it, such as cataracts. The slit-lamp images from cataracts patients show the heterogeneity among different patients, and represent the complexity of ophthalmic images as well. Furthermore, there have been some achievements about automatic diagnosis of ophthalmic diseases have been made, but the researches with slit-lamp images are still relatively less^14^. To fill this gap, common slit-lamp images from patients suffering from pediatric cataract were chosen in our study as research material to explore which methods are effective and efficient in diagnosing ophthalmic disease.

We select some methods which have been applied to diagnose ocular diseases^4^,^9^,^10^,^12^, where Liye Guo *et al*. proposed an entire structure for the automatic diagnosis of cataracts using fundus images. After the features in the images are extracted with wavelet transformation and sketch-based methods, the multiple-fisher classifier divides all of the samples according to the severity of the disease^4^; Muthu Rama Krishnan Mookiah *et al*. proposed an automated dry age-related macular degeneration (AMD) detection system using various features, including entropies, HOS (higher order spectra) bispectral features, fractional dimension (FD), and Gabor wavelet features, which are extracted from grayscale fundus images. The features are then selected with a series of statistical test methods and used to classify with a group of classifiers, such as *k*-nearest neighbor (*k*-NN), probabilistic neural network (PNN), decision tree (DT) and SVMs methods; the average accuracy was greater than 90%^9^; Muthu Rama Krishnan Mookiah *et al*. selected HOS and discrete wavelet transform (DWT) features as image descriptor portraying eye images and SVMs (support vector machines) as classifier to diagnose glaucoma and obtained a higher accuracy than before^10^; Anushikha Singh *et al*. made use of genetic feature selection to choose useful features from wavelet features which is extracted from segmented optic disc images, in which the blood vessels were removed to automatically detect glaucoma with fundus images. Experiment demonstrated that doing so is better than directly classifying with whole or sub-fundus images^12^. Besides, several methods^15^ which have not been employed in this field yet in image classification realm are also selected to try to identify ophthalmic diseases.

Experimental result reveals some methods [e.g. LBP (local binary pattern) +SVMs (support vector machines), wavelet transformation +SVMs] own good performance (recognition accuracy is over 87%). However, although with same features, some methods [e.g. extreme learning machine (ELM), sparse representation] are not suitable for diagnosing ocular disease. Methods with better performance can be adopted to aid doctors in preliminary screening diseases. Furthermore, some methods (e.g. color feature, texture feature combining with SVMs or *k*NN (*k*-nearest neighbor)) requiring less memory or consuming less time can be practically applied in some specific situations, such as mobile devices. In addition, the present study can aid people in remote places or hospitals without advanced equipment in choosing suitable methods for screening ophthalmic diseases.

## Results and Discussion

### Dataset

**Figure 1:**
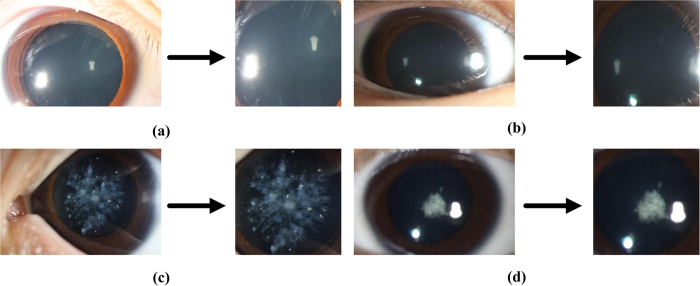
Original slit lamp image and the chosen area for classification.

All of the slit-lamp images in the dataset were obtained from the Zhongshan Ophthalmic Center, Sun Yat-sen University, which is the most advanced ophthalmic hospital in China and sets up a state of the art ophthalmology laboratory^16^. There are 476 slit-lamp images (positive samples) from patients who suffer from pediatric cataracts and 410 slit-lamp images (negative samples) from control individuals. Because cataracts occurs in lens of patients and other parts of eyes is useless for diagnosing this disease, all of the images were manually segmented with tools to allow a rectangle to cover the range of lens and include other parts of the image which is useless for classification as little as possible. The above process will be illustrated in Fig. 1 which provide some examples of slit lamp images and the ROI (region of interest) from them, where Fig. 1(a) and (b) are from normal people and Fig. 1(c) and (d) are from patients.

### Experimental settings and performance evaluation indicator

Eight classification schemas are shown in Table 1 along with the parameters of them. Four-fold cross validation was adopted to permit a fair comparison of these methods. Eight groups of feature extraction algorithms and image classification methods are assembled to automatically diagnose pediatric cataracts, and their performances were compared in various respects. All schemas are divided in three groups: the feature extraction methods in the first group [schema (1), (2), (3) and (4)] is color features combined with texture features and the classification methods are different, subsequently the best classifier in these four schemas can come into being; the second group [schema (5) and (6)] combines better features and the best classifier which is obtained in the first group so that the best schema will be obtained; the last, sparse representation which has been rarely used to recognize medical images is used to identify ocular images in the third group [schema (7) and (8)]. The eight schemas were implemented with MATLAB R2014a on a personal computer with an Intel 3.60 GHz i7 processor with 16 G RAM. The objective of our study is to apply these methods to automatic identifying of ophthalmic disease, validation of their feasibility and comparison of the performance of these methods; the performance indicators that were employed to evaluate the performance are as follows:


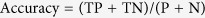



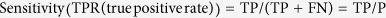



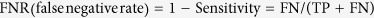



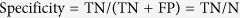



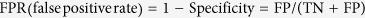


where P and N are the number of positive samples and negative samples respectively; TP indicates the number of positive samples which are classified into the positive class; FN denotes the number of positive samples classified as negative samples; TN is the number of negative samples recognized as negative samples; and FP refers to the number of negative samples that are identified as positive samples. Besides, ROC (receiver operating characteristics) curve, which indicates how many positive samples are recognized conditioned on a given false positive rate and AUC (area under the curve) which means the area of the zone under ROC curve are also adopted to assess the performance.

### Comparison of the performance of schemas (1), (2), (3) and (4)

**Figure 2:**
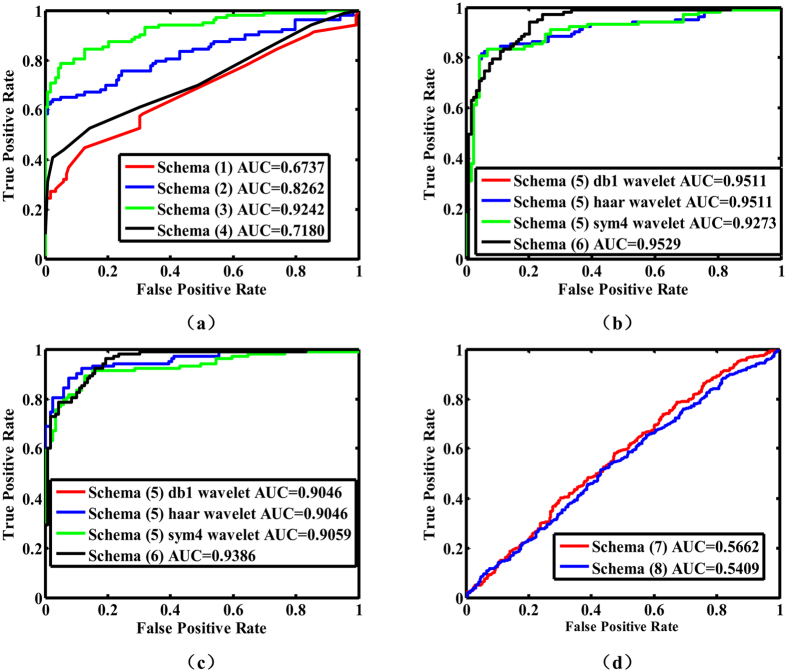
ROC curves for eight schemas. This figure shows the ROC curves of eight schemas (**a**) is the ROC curves of schema (1), (2), (3) and (4), where the *k* of schema (4) is 10; (**b**) shows the ROC curves of schema (5) and (6). Schema (5) contains the discrete wavelet transformation with three different wavelets which is used as a method to extract image feature. So there are three different curves about schema (5), where two curves are coincident. The kernel function in SVMs (support vector machines) of these two schemas is linear kernel function; (**c**) shows the ROC curves of schema (5) and (6). Schema (5) contains the discrete wavelet transformation with three different wavelets which is used as a method to extract image feature. So there are three different curves about schema (5), where two curves are coincident. The kernel function in SVMs of these two schemas is polynomial kernel function; (**d**) displays the ROC curves of schema (7) and (8). The over-complete dictionary of schema (7) is formed with 1 negative sample and 80 positive samples, whereas that of schema (8) is formed with 5 negative samples and 70 positive samples (the sample size in schema (6) is 5 × 10). All sub-images are from the first-fold out of four-fold cross-validation. (ROC: receiver operating characteristics curve; AUC: area under the curve).

First, the performance of schemas (1), (2), (3) and (4) are compared. As is shown in Table 2, the format of these data is *μ* ± *δ (μ* is the mean value, *δ* is standard deviation). Since FNR and FPR could be computed with sensitivity and specificity, all FNR and FPR are not shown. The linear kernel was chosen as the kernel function in schema (2) and (3), because the SVMs with other kernel functions could not converge within an acceptable time. Schema (3) demonstrated highest accuracy among these four schemas in terms of recognition accuracy, and compared with schema (2), the classification accuracy of schema (3) which select a part of features that are helpful for diagnosis of ocular images has been improved significantly. Besides, the performance of schema (1) is unsatisfactory and schema (4) performs relatively satisfactory no matter how great the parameter *k* is. Then the overall performance of these four schemas was further assessed by using ROC curve and AUC values which are shown in Fig. 2(a), where the *k* of schema (4) is 10. GA (genetic algorithm) is a probabilistic algorithm so that the results obtained by schema (3) may be different each time, thus schema (3) is repeated ten times and the ROC curve results from highest accuracy, which shows that three color features and seventeen texture features are helpful for classification and is shown in Fig. 2(a). All of the above results indicate that schema (3) clearly demonstrates the highest accuracy in these four schemas.

### Comparison of the performance of schemas (5), and (6)

Because the performance of different classifiers with same image features has been compared in above comparison stage, SVMs is selected to be the classifier in this comparison stage. In schema (5), all of the wavelet coefficients were obtained using two-level discrete wavelet transformation originating from three different types of wavelet. The performance of schema (5) with the linear kernel is almost as good as that using the polynomial kernel, while the performance of schema (5) using other kernel functions was not desirable. A performance comparison for schemas (5) and (6) with two types of kernel functions is shown in Table 3, which indicates the two schemas are good at identifying ophthalmic images. The ROC curves and AUC values for schemas (5) and (6) with different kernel functions are shown in Fig. 2(b) and (c). Compared with the other schemas, schemas (5) and (6) demonstrate the most outstanding robustness and performance. On the other hand, this result also indicates that the feature extraction procedure of schemas (5) and (6) was able to exactly depict the special characteristics of samples belonging to two different classes and the features from them subsequently facilitate the classification. Because the ROC curves of schemas (5) and (6) are similar, which is not helpful for comparing their performance, the AUC values were used for a more objective and precise comparison. As is shown in Fig. 2(b) and (c), LBP is more suitable than wavelet in depicting image features in terms of the AUC values.

### Comparison of the performance of schemas (7), and (8)

In schema (7), the sample size (image size) is different for different application, so the sample size is investigated in experiment to find how it affect the classification accuracy. Table 4 shows the performance of schema (7) and schema (8). It is found that schema (7) was able to achieve better performance with smaller sample size while schema (8) performs relatively poorly in solving this problem. Thus, we attempted to establish a set of proper parameters for schemas (7) and (8).

We use an over-complete dictionary formed with fewer images and all of the remaining images not included in the over-complete dictionary were chosen as testing samples. The number of positive samples in the dictionary is larger than that of negative samples, which is expected because negative samples are quite similar, whereas the conditions of positive samples are very complicated. That’s to say, it is not necessary to use more negative representative samples that are to be classified. The performance of schema (7) in which all images are resized to be uniform size (15 × 20 or 5 × 10) and schema (8) can be improved by using an over-complete dictionary with the appropriate size. Schema (7) also can obtain better performance with a proper sample size. The performance of schemas (7) and (8) with different parameters are shown in Table 5 which illustrates that compared with the original schemas (7) and (8), the performance of the schemas (7) and (8) with different parameters was enhanced. Furthermore, the performance of schema (7) is slightly better with a smaller sample size rather than a larger sample size, which is verified in Table 5. Then compared with schema (4) whose features are same with schema (8), the performance of schema (8) is unacceptable, which signifies sparse representation is unsuited to solve this task.

The ROC curves for schema (7) with one negative sample and 80 positive samples and for schema (8) with five negative samples and 70 positive samples (all of the samples are resized to a uniform size (5 × 10)) are shown in Fig. 2(d). Though the classification accuracy of schemas (7) and (8) are able to surpass that of schema (1) when proper parameters are utilized, the AUC values of schemas (7) and (8) are smallest in these eight schemas. Furthermore, the AUC values of schema (7) and (8) are so close to 0.5, which indicates that the classification nearly becomes randomly guessing without any reason.

### Comparison of computational and memory complexity of all schemas

**Figure 3:**
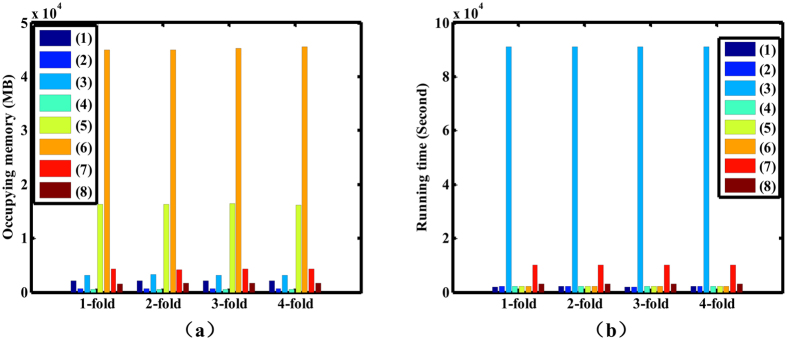
Memory usage and Computational time of the 8 schemas. This figure is the memory space occupied by all schemas and running time of these schemas in identifying ophthalmic disease. (**a**), occupied memory of all schemas. (**b**), running time of all schemas. (MB: Megabyte).

Finally, the memory usage which is shown as Fig. 3(a) and running time which is shown as Fig. 3(b) were used to evaluate the memory and computational complexity of the schemas. Because the classification of 4-fold cross validation is combined together in schema (3), the memory usage and running time of schema (3) are divided into four equal parts. The running time and memory usage of schemas (5) and (6) with different kernel functions and different wavelets are almost the same, thus the mean values are shown in Fig. 3. The performance indicators for schema (7) and (8) with modified parameters (5 negative samples, 75 positive samples and 5 × 10 sample size for schema (7); 1 negative samples and 80 positive samples for schema (8)) are shown in Fig. 3. The relevant data for schema (4) with *k* = 10 are shown in Fig. 3. There are some significant differences between the eight schemas in memory usage. Schemas (5) and (6) occupy more memory than the other schemas, but the performances of schemas (5) and (6) are excellent, so it is worth exchanging some memory space for high performance and accuracy. Schema (2) can achieve satisfactory performance with little memory space within a little time. Compared with schemas (1), (2) and (4), schema (7) was unable to provide a relatively good result with occupying a comparatively larger memory space and consuming a relatively longer time. Although schema (8) does not consume as much resources as schema (7), the result provided by schema (8) is not satisfactory either so no meaningful diagnosis advices could be provided by schemas (7) and (8). In terms of running time, the method that requires the longest time is schema (3) whose loss outweighs the gain in some extent. Except for schema (3), the computational time of the remaining schemas are reasonable in terms of computational complexity.

## Conclusions and Future Work

Eight groups of representative methods for image feature extraction and image classification have been applied to automatically identify ophthalmic images. In present study, the performance of the eight schemas are compared with regard to multiple aspects. No matter which feature extraction method is adopted, SVMs (support vector machines) classifier yielded desirable performance, which verified by four schemas (schemas (2), (3), (5), (6)). Some traditional methods, such as the *k*NN (*k*-nearest neighbor) approach, were also able to provide relatively satisfactory results. Although the ELM is quicker than other neural network architectures when applied to classification, the results returned from it were undesirable. Similar to the ELM (extreme learning machine), sparse representation is not good at solving this problem and the accuracy of sparse representation with modified parameters are improved insignificantly. This problem may be solved by using an advanced or improved optimization algorithm^17^ and an excellent dictionary learning algorithm^18^, which is promising in the field of image classification. At the same time, the feature extraction methods, such as wavelet transformation, the LBP (local binary pattern) algorithm, color features and texture features, were able to express the image characteristics in varying degrees to facilitate classification. In the future, efforts can be made to explore other image feature extraction methods and image classification algorithms, such as deep learning which finishes feature extraction and classification meanwhile and thus suited to solve this problem and obtain a better performance. At last, those algorithms that own better performance and do not need much computational resource could be used to help doctors screen ophthalmic diseases or to help individuals eliminate these diseases. Some methods could be deployed in remote places without advanced equipment or hospitals without experienced doctors. Furthermore, methods with better performance could be deployed on mobile phones without large capacity memory or high-speed processors to help individuals take precautions against ophthalmic diseases.

## Methods

### Color features extraction

The pictures are formed with pixels that express the color features^19^, which can provide valuable statistical information about the color distribution of a picture; thus, color features is an important category of image features. Color features are applied in image segmentation problems^19^, image classification problems^20^ and so on. Color features extraction should be based on a specific color space, such as HSV (hue, saturation, value), HSI (hue, saturation, intensity) or RGB (red, green, blue). The RGB color space is chosen in the present study to determine the first-, second- and third-order moments of 3 channels, as is shown in equations (1, 2, 3).


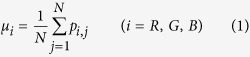



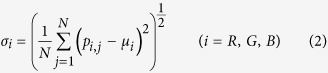



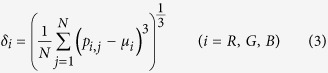


where *N* is the total number of pixels in an image and *p*_*i,j*_ is the level of the R, G or B component in one pixel. Nine color features are analyzed in equations (1, 2, 3).

### Texture features extraction

Texture features are used to depict the variation relations among different pixels in an image. Gray tone spatial dependence matrices and gray gradient co-occurrence matrices are employed to extract the texture features of an image and have been applied in many fields, such as automatic medical diagnosis^21^,^22^, defect detection^23^ and pattern classification^24^.

As a typical texture feature extraction method, statistic-based gray tone spatial dependence matrices^25^ can present the comprehensive information of the gray distribution in terms of different aspects, including direction, variation range and local domain. The elements in gray tone spatial dependence matrices are defined as the occurrence frequency of gray values of 2 different pixels separated by *d* pixels in direction *θ* and whose gray values are *i* and *j*, respectively. θ is generally set as 0°, 45°, 90° or 135°; therefore, the 4 matrices whose elements are shown in equations (4,5,6,7) can be obtained with this method. A succession of features can be computed with gray tone spatial dependence matrices and can be used to form part of a whole feature vector in the present study.


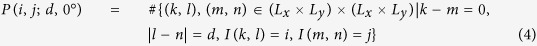



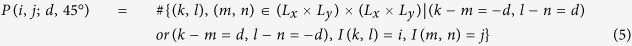



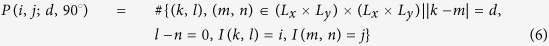



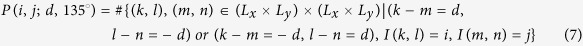


where (*k, l*) and (*m, n*) are pixel coordinates in the images; *I(k, l*) and *I(m, n*) are the gray values of the corresponding pixels; *L*_*x*_ and *L*_*y*_ are the numbers of rows and columns of the gray tone spatial dependence matrices respectively, which are related to the level of gray. The summation of the four gray tone spatial dependence matrices is then normalized to compute 14 texture features. The # symbol denotes the number of elements in a set.

Compared with the gray tone spatial dependence matrices, the gray gradient co-occurrence matrix^26^ containing both the variation of gray and the gradient is also commonly employed to represent the texture features of an image. The 15 features computed with the gray gradient co-occurrence matrix are also chosen as a part of the feature vector of the images. The gradient image *G* is calculated, and its elements are dispersed into *L*_*g*_ gray levels. Subsequently, the gray gradient co-occurrence matrix can be built with *G* and the original images *I* using equation (8).


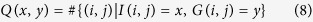


where (*i, j*) is the pixel coordinate in the image; *I(i, j*) and *G(i, j*) are the gray values of corresponding pixels in *I* and *G* respectively. The # symbol denotes the number of elements in a set.

### Wavelet transformation

The wavelet transformation^27^, which can be used to effectively analyze the features of an image at different scales, has been frequently applied in the field of image processing and image classification^27^,^28^. The wavelet transformation provides the time-frequency information of the images, which is a preferred method for replacing some shallow time invariant image features.

Given *f(x*), a continuous, square-integrable function, the continuous wavelet transformation of *f(x*) is


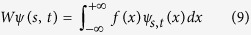


where *ψ*_*s,t*_(*x*) is constructed from real-valued mother wavelets with different scale factor *s* and different transition factor *t*.

If *s*_*0*_ is a proper value such that 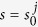, where *s*_*0*_ > 0 is an expansion step; when the scale factor *s* = *s*_*0*_ and the translation factor *t* = *t*_*0*_, each translation can be *kt*_*0*_. For 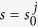, 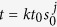, *k, j* *∈* *Z*, and *s*_*0*_ > 1, *t*_*0*_ > 1 are constants, and the discrete wavelet is derived as equation (10):


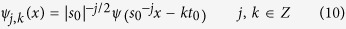


Thus, the corresponding wavelet transformation is given as equation (11):


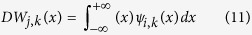


The wavelet coefficients extracted from images using equation (11) can be regarded as feature vectors and forwarded into the classifier to identify pediatric cataracts.

### LBP (local binary pattern)

The powerful LBP^29^,^30^ not only characterizes rotation invariance, gray invariance and other elements but is also a simple yet effective operator depicting the local texture feature of images; the LBP operator summarizes the relationship between the gray value of the central pixel and the gray values of its neighbors, which is refined as equation (12). The gray intensity of each surrounding pixel is compared with the gray intensity of the central pixel. If the gray value of the surrounding pixel is larger than or equal to the gray value of the central pixel, the corresponding bit of the LBP code is assigned 1; otherwise, the corresponding bit of the LBP code is assigned 0. The LBP code can be expressed as equation (12), where *g*_*c*_ and *g*_*i*_ denote the gray value of the central pixel and the gray value of a neighboring pixel surrounding the central pixel, respectively. Here *P* = 8 and *s*(·) is defined as in equation (13):


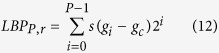



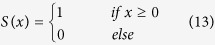


If equation (12) is exploited to calculate the LBP code, 256 different LBP codes will arise in the LBP descriptor of the image. The fact that some special patterns known as uniform patterns emerge more frequently than remaining type of patterns can help to drastically reduce the number of LBP codes. If the total number of transitions from 1 (0) to 0 (1) in an LBP code is less than 2, this LBP code is classified as a uniform pattern. The LBP value of a pixel can be computed with this extended LBP operator; see equation (14). The LBP feature is created by summarizing the histogram of LBP values in each region of the partitioned image and then connecting these histograms.


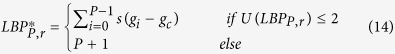


where *U(LBP*_*P,r*_) is the total number of transition from 1 (0) to 0 (1). *s*(·) is given as equation (12).

### SVMs (support vector machines) classifier

Statistical learning-based SVMs^31^, which apply structural risk minimization theory, have emerged as a new pattern classification method in recent years and exhibits strong generalization. SVMs demonstrates high performance, owns reliable theoretical support and have been applied in many fields^32^,^33^,^34^. It is utilized to solve binary classification problem at first. Given a dataset in which the samples belong to one of two classes, the SVMs attempt to find a hyperplane to separate samples from the two different categories and to maximize the distance between the 2 different classes. Given *i* pairs of training data from two classes 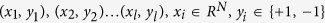, where *x* and *y* are the feature vector and label of one sample respectively, the decision surface of linear separable data can be expressed as equation (15):


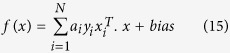


In this case, *a*_*i*_ is a Lagrange multiplier, the vectors corresponding to *a*_*i*_ > 0 are supporting vectors, and *f(x*) is not affected by the dimension of the feature space. The decision surface of the non-linear separable data can be presented as equation (16):


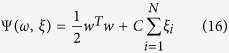


For the non-linear separable problem, the kernel function mapping low-dimension data into high-dimension space can define a new data point corresponding to the old data point in a new feature space. Because the type of kernel function is not unique, the choice of kernel function should depend on specific conditions. The LIBSVM^35^ is adopted to carry out the experiments relevant to SVMs in the present study.

### ELM (extreme learning machine) classifier

The ELM^36^, which does not require gradient information of error function and only needs to solve a matrix equation rapidly during its training process, is an improved neural network architecture. Suppose there are *N* samples and a three-layer feedforward neural network, whose hidden layer contains 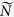
neurons. If *g(x*) is the activation function of neurons in the hidden layer, then the output matrix of the hidden layer is 
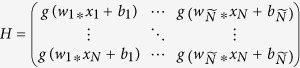
. The weight matrix between the hidden layer and the output layer is 
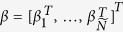
, The output matrix of the output layer is 
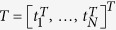
. The classification problem can be completed by solving the matrix formula *Hβ* = *T*, namely, *β* = *H*^+^*T*, where *H*^+^ is the generalized inverse of *H*.

### Sparse representation

Sparse representation^37^,^38^ is an effective method converting classification problem into an optimization problem. Firstly, an over-complete dictionary is constructed using a portion of the images, and the remaining images that are not included in the over-complete dictionary are represented by the images in the over-complete dictionary. Suppose that some samples belong to *k* classes and the training samples belong to the *i*th class constitute 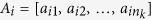 ∈ 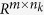; then training samples belonging to *k* classes can be combined as the over-complete dictionary A = [A_1_, A_2_,…,A_k_] ∈ 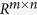, and a sample *y* to be classified can be presented as equation (17):


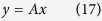


where x is the coefficient vector, which can be understood as the projection of y on *A*. Ideally, the coefficients corresponding to the *i*th class, which is the real class of *y*, are non-zero, and the remaining coefficients are zero. If *n* is large enough, then *x* is sparse. If *m* < *n*, this problem will convert into a minimization problem of the *L*_*0*_ norm, namely,


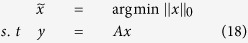


Because this problem is NP hard, according to compressed sensing theory, this problem can be converted as follows:


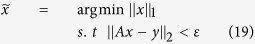


To solve equation (19), The differential evolution (DE) algorithm^39^, whose key procedures include differential mutation, crossover and greedy choice, is introduced. The differential mutation and crossover should be implemented as equations (20) and (21), respectively.


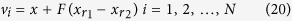


where *v*_*i*_ is the differential vector created from equation (20) and *x*, 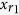, 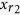 and 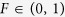 are the aim vector, first randomly selected vector, second randomly selected vector and scaling factor respectively. *N* is the size of the population.


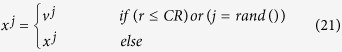


where *x*^*j*^ is the *j*th component of the cross vector corresponding to the selected aim vector, 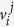 is the *j*th component of the differential vector and 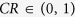 is the cross factor.

Because this problem is a constrained optimization problem, the Deb criterion is introduced into the greedy choice of DE as follows. If both solutions are in feasible region, choose the individual with the higher fitness; if neither of the two solutions is in feasible region, choose the individual that violates constraints less; if one of the solutions is in feasible region and the other solution is not, choose the individual in the feasible region.

### Feature selection

Because the high dimension of the feature vector of the images leads to difficulty in distinguishing which feature is helpful for classification, the genetic algorithm (GA)^40^,^41^ and SVMs (support vector machines) are combined to solve the image classification problem, which will implement feature selection and classification at the same time. The accuracy of the SVMs is adopted as the fitness evaluation function of the GA. The chromosome coding method is binary coding; the length of the chromosome equals the dimension of the feature vector; and a bit 0 signifies that the feature corresponding to this bit is not needed in classification; otherwise, the feature is needed in classification.

### *k*NN (*k*-nearest neighbor) classifier

*k*NN^42^,^43^ is a simple yet well-known classification approach that uses information from the *k* closest data points for classification. This algorithm makes use of some distance measurements to compare the distance or similarity between two data points, such as the Euclidean distance, Manhattn distance and Canberra distance. The labels of the data point to be classified should be consistent with the labels of the majority surrounding this data point. The *k*NN approach has been applied in many fields, such as big data^42^ and biology^43^.

## Additional Information

**How to cite this article**: Wang, L. *et al*. Comparative analysis of image classification methods for automatic diagnosis of ophthalmic images. *Sci. Rep.* **7**, 41545; doi: 10.1038/srep41545 (2017).

**Publisher's note:** Springer Nature remains neutral with regard to jurisdictional claims in published maps and institutional affiliations.

## Figures and Tables

**Table 1:** The relevant parameters of eight schemas.

Schema	Feature extraction	Classification
(1)	Color features; gray tone spatial dependence matrices: *d* = 1; gray gradient co-occurrence matrices: *L*_*g*_ = 10	ELM (extreme learning machine): The number of neurons in hidden layer is 80
(2)		SVMs (support vector machines): Linear kernel function
(3)		SVMs: Linear kernel function; GA (genetic algorithm): population size is 30; crossover rate is 0.7; mutation rate is 0.3; maximum iteration steps is 200
(4)		*k*NN (*k*-nearest neighbor): *k* = 5, 10, 20
(5)	Wavelet transformation: Two level wavelet transformation; three kind of wavelets: db1, sym4, haar; all images are resized to be 15 × 30	SVMs: Linear kernel function; polynomial kernel function
(6)	LBP (local binary pattern): *P* = 9; the size of each window is 9 × 9; all images are resized to be 20 × 30	
(7)	Sparse representation: Sample size: 15 × 20, 5 × 10; size of over complete dictionary: 70, 75, 78, 80, 81, 85, 90	DE (differential evolution): Population size is 50; crossover rate is 0.7; mutation rate is 0.4; maximum iteration steps is 500
(8)	Color features; gray tone spatial dependence matrices: *d* = 1; gray gradient co-occurrence matrices: *L*_*g*_ = 10 sparse representation: size of over complete dictionary: 70, 80, 81, 85	

**Table 2:** Performance comparison of schemas (1), (2), (3) and (4) with different *k*.

Schema		Accuracy	Sensitivity	Specificity
(1)		0.5825 ± 0.0336	0.3123 ± 0.0853	0.8151 ± 0.0227
(2)		0.7844 ± 0.0337	0.5610 ± 0.0585	0.9769 ± 0.0144
(3)		0.8341 ± 0.2788	0.7074 ± 0.0822	0.9433 ± 0.0325
(4)	*k* = 5	0.6976 ± 0.0241	0.5877 ± 0.0391	0.7829 ± 0.0603
	*k* = 10	0.7133 ± 0.0198	0.5732 ± 0.0347	0.8340 ± 0.0403
	*k* = 20	0.7100 ± 0.0293	0.5315 ± 0.0118	0.8550 ± 0.0359

**Table 3:** Performance comparison of schemas (5) and (6).

Schema	Feature extraction method	Kernel function	Accuracy	Sensitivity	Specificity
(5)	haar wavelet	linear	0.8758 ± 0.0315	0.7976 ± 0.0517	0.9432 ± 0.0186
		polynomial	0.8814 ± 0.0109	0.7853 ± 0.0263	0.9642 ± 0.0186
	db1 wavelet	linear	0.8758 ± 0.0315	0.7976 ± 0.0517	0.9432 ± 0.0186
		polynomial	0.8814 ± 0.0109	0.7853 ± 0.0263	0.9642 ± 0.0186
	sym4 wavelet	linear	0.8679 ± 0.0288	0.7902 ± 0.0589	0.9348 ± 0.0210
		polynomial	0.8611 ± 0.0104	0.7463 ± 0.0249	0.9579 ± 0.0194
(6)	LBP	linear	0.8635 ± 0.0248	0.8367 ± 0.0404	0.8865 ± 0.0223
		polynomial	0.8827 ± 0.0189	0.8463 ± 0.0289	0.9118 ± 0.0287

**Table 4:** Performance of schemas (7) and (8).

Schema	Sample size	Accuracy	Sensitivity	Specificity
(7)	5 × 10	0.5282 ± 0.0260	0.5552 ± 0.3777	0.5063 ± 0.3673
	15 × 20	0.4627 ± 0.0014	1 ± 0	0 ± 0
(8)	—	0.4790 ± 0.0061	0.8900 ± 0.0851	0.0996 ± 0.1006

**Table 5:** Performance of schema (7) using an over-complete dictionary with different sizes and different sample sizes and schema (8) for different over-complete dictionary sizes.

Schema	Image size	Number of images belonging to each class in the over-complete dictionary		Accuracy	Sensitivity	Specificity
		Negative samples	Positive samples			
(7)	15 × 20	20	50	0.5623	0	0.9910
		20	60	0.5757	0.0147	0.9850
		15	70	0.5808	0.0088	0.9936
		10	70	0.5845	0.0942	0.9384
		5	70	0.5931	0.1059	0.9488
	5 × 10	10	80	0.5792	0.0303	0.9678
		10	75	0.5730	0.0746	0.9313
		3	75	0.5916	0.1400	0.0888
		5	75	0.5943	0.1224	0.9299
		5	70	0.5993	0.1882	0.8960
(8)	—	20	50	0.5732	0.0265	0.9721
		10	70	0.5734	0.0265	0.9682
		10	75	0.5796	0.0091	0.9768
		5	75	0.5801	0.0515	0.9474
		1	80	0.5826	0.0636	0.9432
